# Supplementary material for: The relationship between regulatory changes in cis and trans and the evolution of gene expression in humans and chimpanzees
Source: Genome Biol. 2023 Sep 11;24:207. doi: 10.1186/s13059-023-03019-3 (PMC10496171; doi:10.1186/s13059-023-03019-3)
Supplement: Supplementary file 1 — Additional file 1. Supplementary figures, tables, and detailed description of Additional files 2, 3, and 4. [file 13059_2023_3019_MOESM1_ESM.docx]

**Additional Files**

**Figures S1 to S10**

**
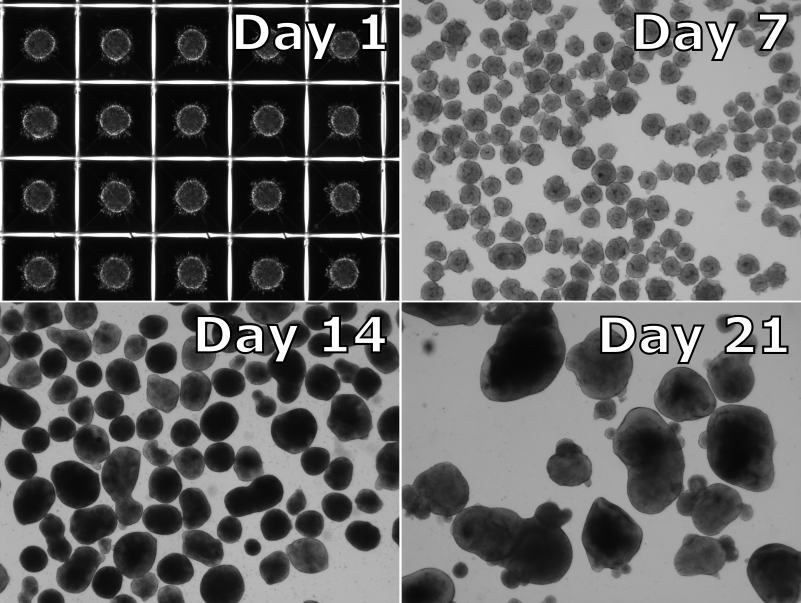
**

**Figure S1.** **Representative images of embryoid bodies.** Images of EBs at days 1, 7, 14, and 21 post-formation. Images are taken on an EVOS 2 FL at 4x resolution.

**
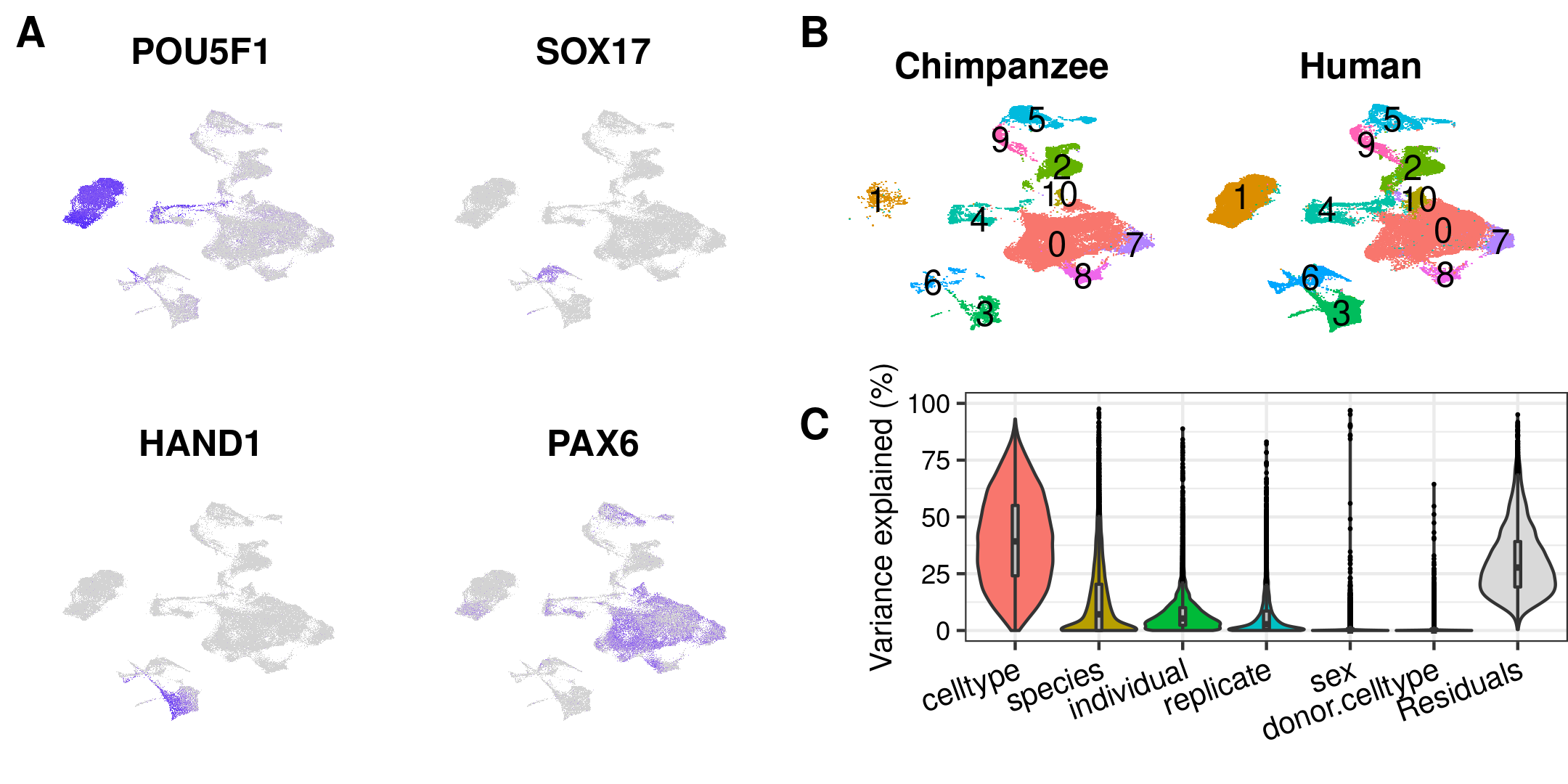

Figure S2.** **Cell type composition in embryoid bodies without external reference integration.** (**A**) UMAP of EB cells highlighting expression of germ layer markers *POU5F1*, *SOX17*, *HAND1*, and *PAX6*. (**B**) UMAP split by species. Cells are colored by Seurat clusters at resolution 0.1. (**C**) The percent of variance explained by biological and technical factors using the VariancePartition R package. Celltype refers to the clusters illustrated in Figure S2B. Donor cell type specifies whether the iPSCs were derived from YRI LCLs or CEU fibroblasts.


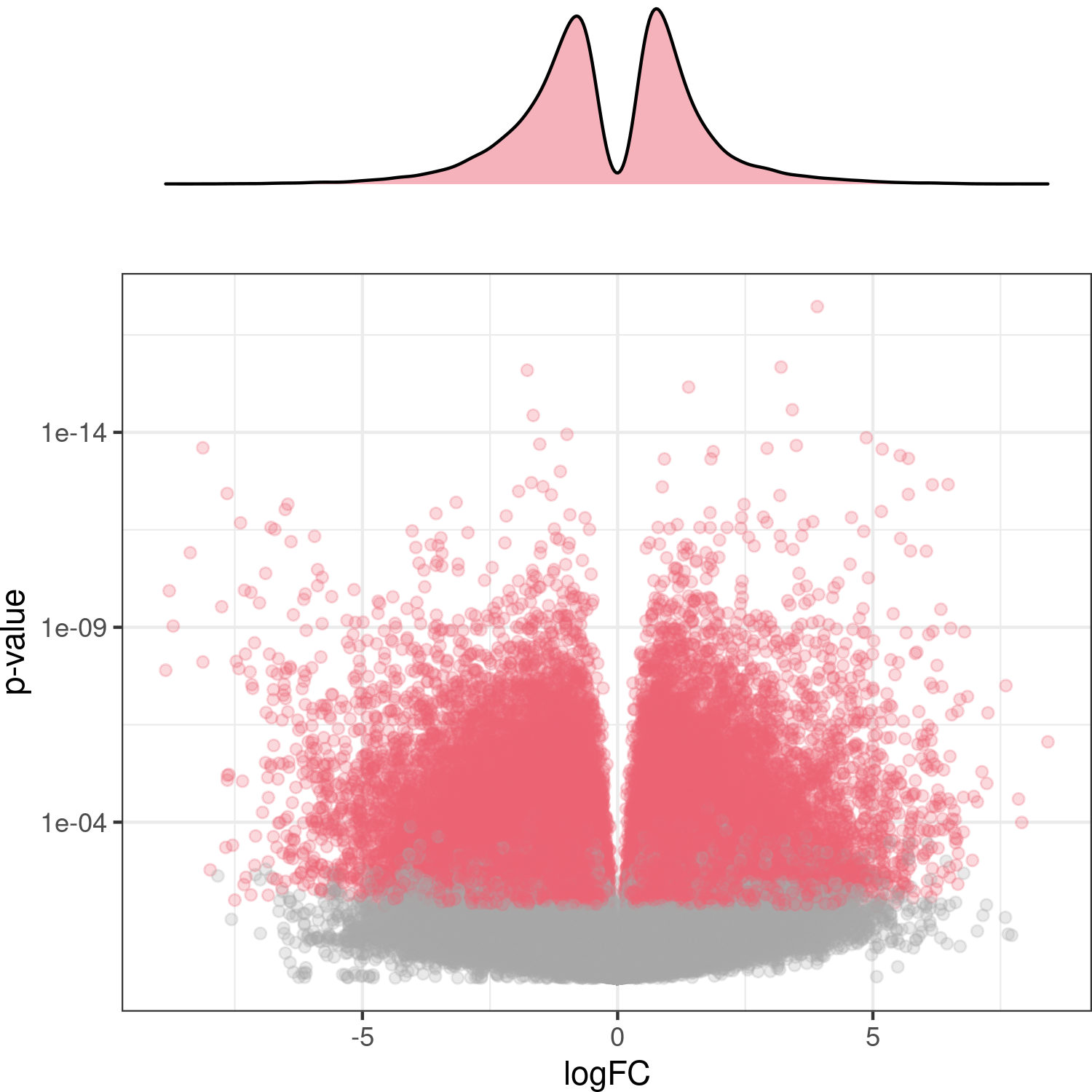


**Figure S3.** **Volcano plot of DREAM statistics.** The nominal *p*-value is plotted as a function of effect size estimate (log2 fold-change gene expression) for all tests across all cell types. We controlled FDR at a 5% level within each cell type. Tests that are significant at this level are colored red. The distribution in effect sizes for significant tests is plotted above the volcano plot.

**
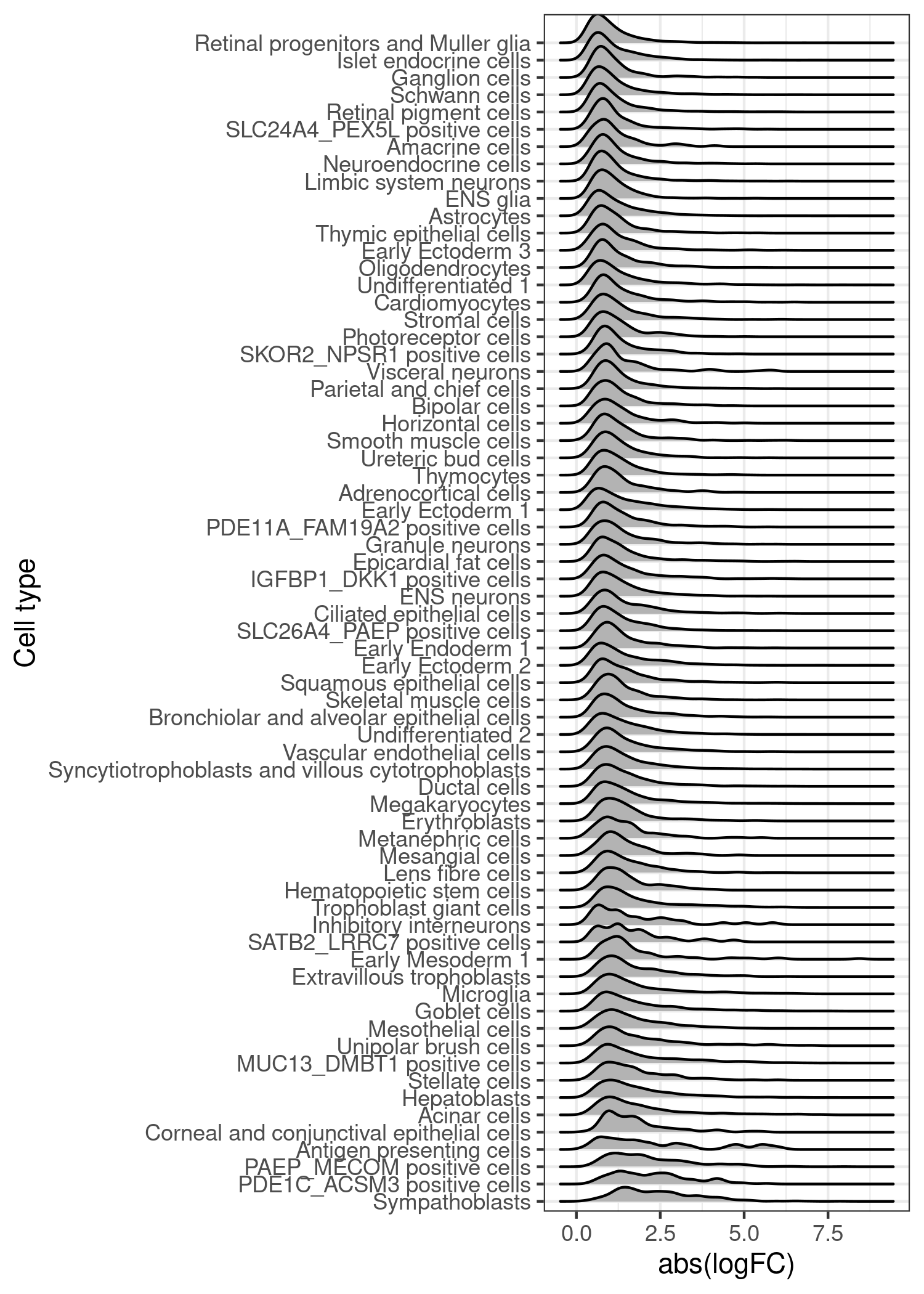
**

**Figure S4. Ridge plot showing distribution of effect sizes in each cell type.** The density in the effect size estimate (log2 fold-change gene expression computed with DREAM) for DE genes is shown in each cell type. We excluded cell types with fewer than 10 DE genes.


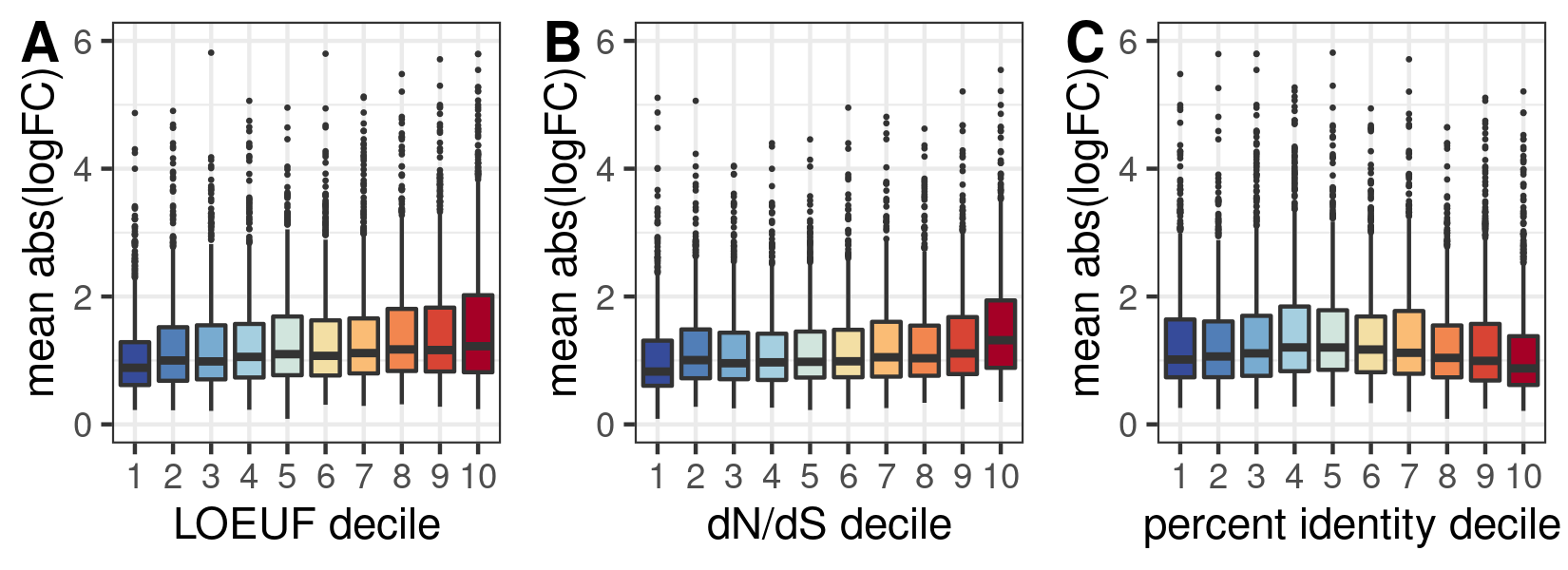


**Figure S5. Association of logFC with LOEUF, dN/dS, and percent coding identity.** For each gene we calculated the mean absolute log2 fold-change in expression. This change significantly associates with **(A)** LOEUF decile (ρ = 0.16; p < 2 x 10^-16^), **(B)** dN/dS decile (ρ = 0.15; p < 2 x 10^-16^) and **(C)** percent coding identity decile (ρ = -0.066; p = 2.46 x 10^-11^).

**
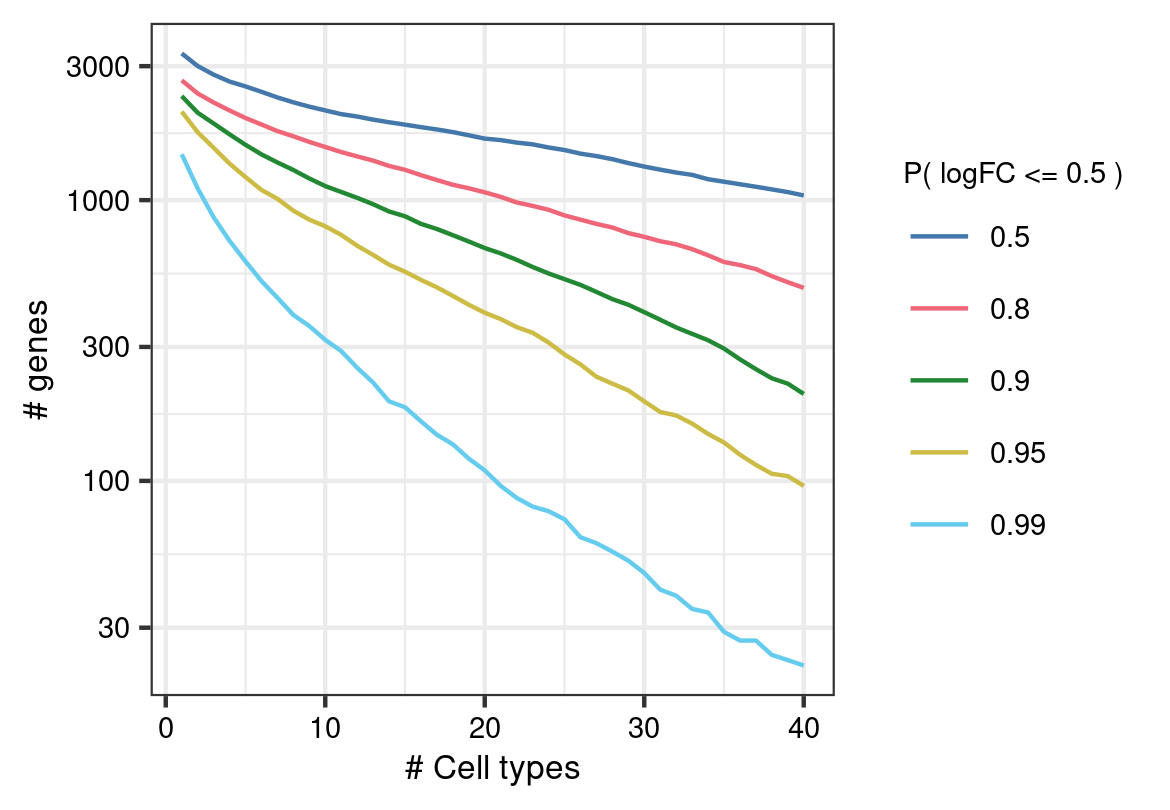
**

**Figure S6. Number of genes with small effect sizes across cell types.** We computed the probability that the absolute logFC difference in gene expression between humans and chimpanzees was less than 0.5. We removed genes that had an effect size larger than 0.5 in any cell type (*p* ≤ 0.05). We show how the number of genes with small effect sizes varies with the confidence in this estimate as well as the number of cell types in which the effect size is small.


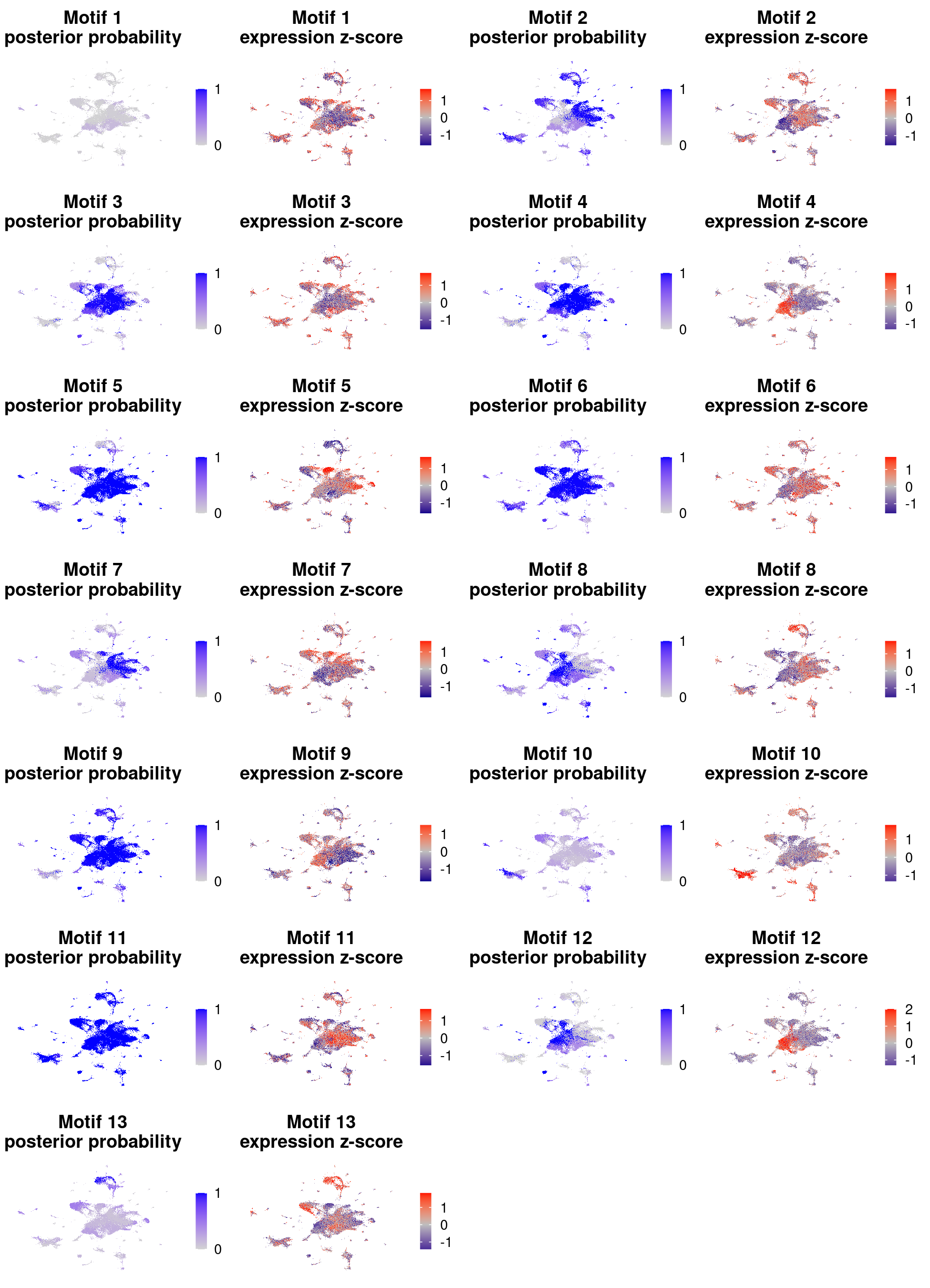


**Figure S7. UMAPs of Cormotif posterior probability of differential expression and gene expression z-scores.** UMAPs from **Figure 1B** with cells colored according to their posterior probability of DE or gene expression z-score (see Materials and methods).

**
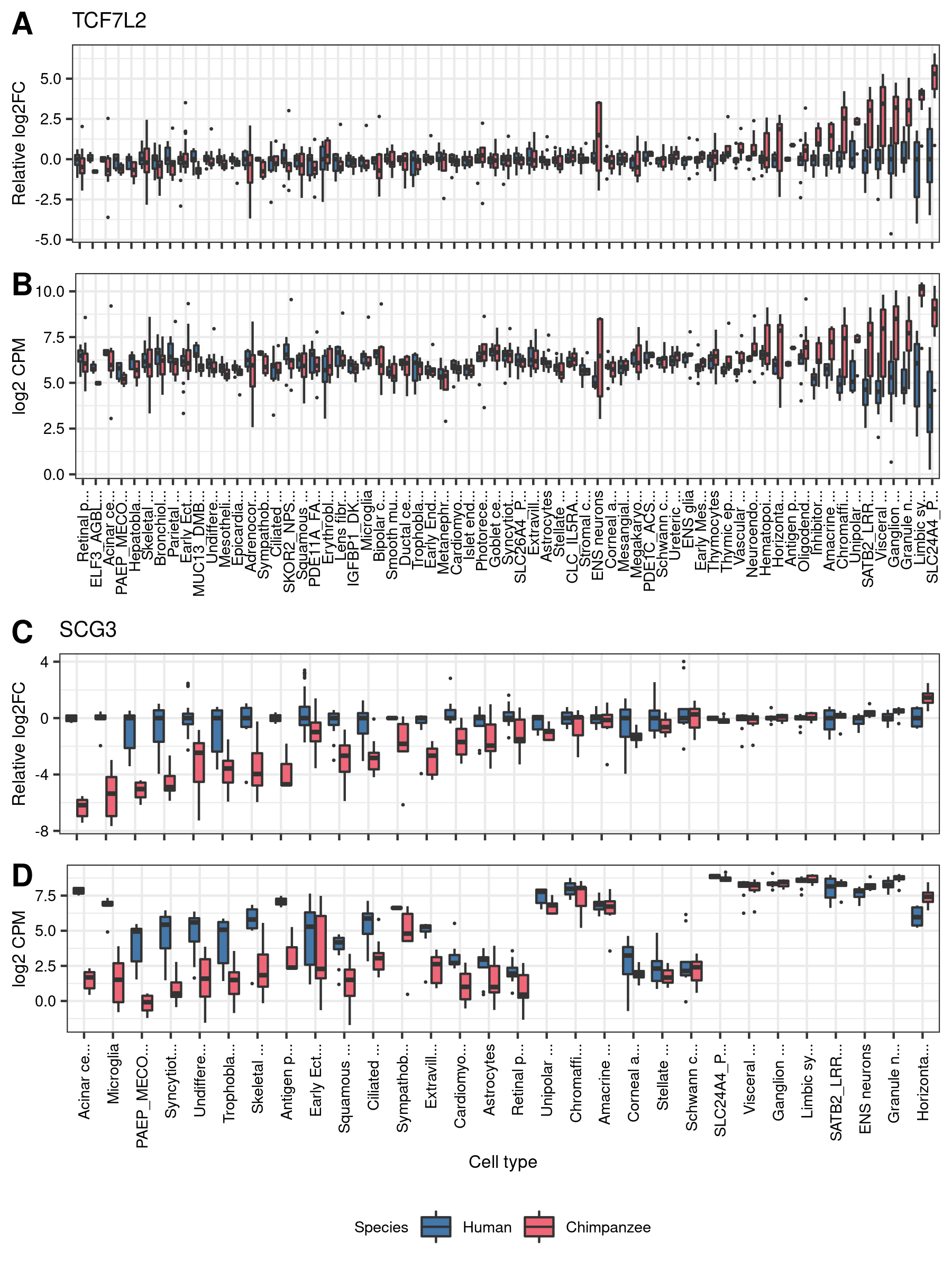
**

**Figure S8. Boxplots of *TCF7L2* and *SCG3* expression.** (**A**) Expression of *TCF7L2* in all tested cell types, relative to the mean expression in humans. (**B**) Expression of *TCF7L2* in all tested cell types. (**C**) Expression of *SCG3* in all tested cell types, relative to the mean expression in humans. (**D**) Expression of *SCG3* in all tested cell types.


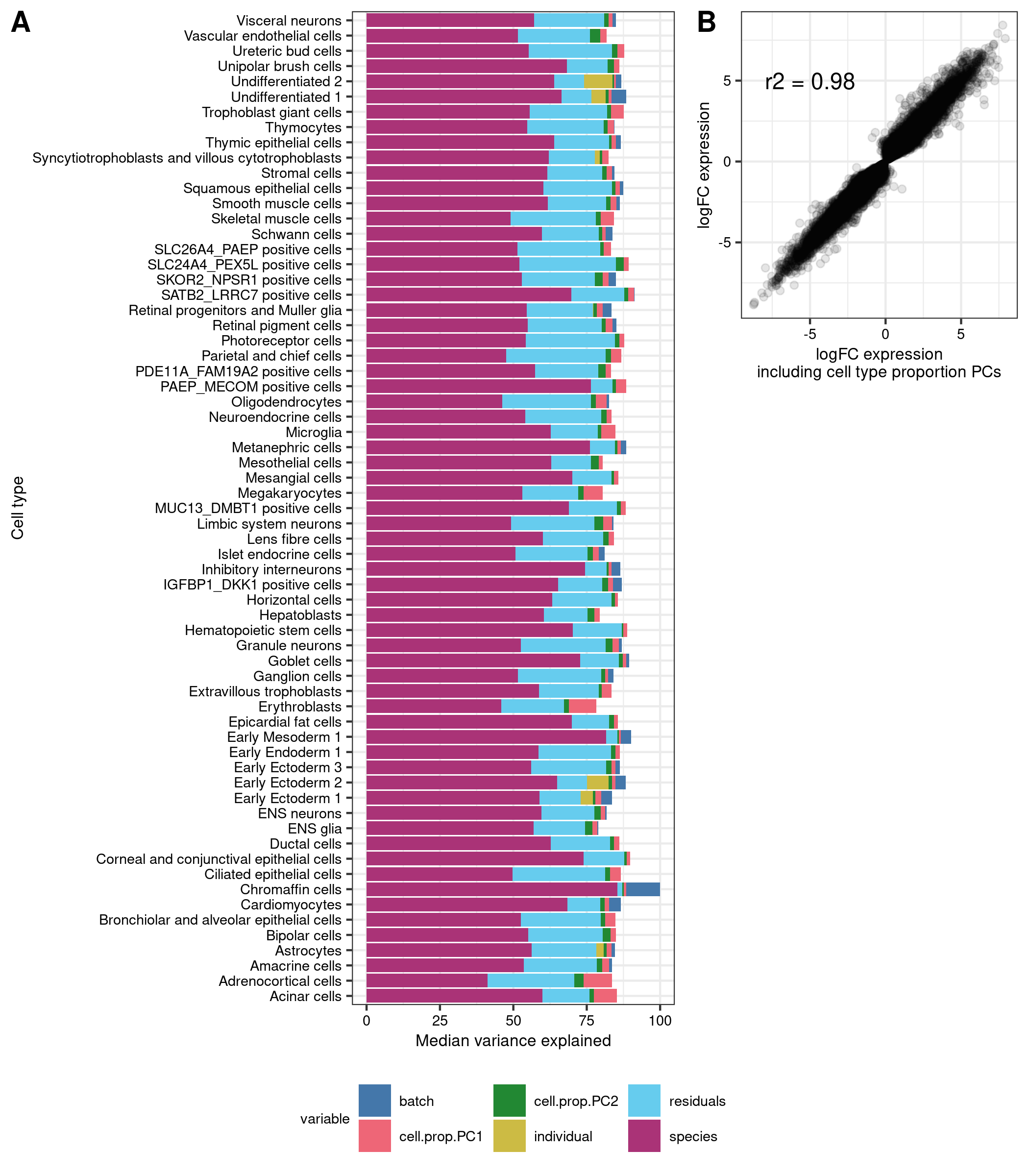


**Figure S9. Effect of cell type proportion.** We ran a linear mixed model including two cell type proportion PCs in DREAM. We then assessed the effect of these PCs on genes with significant DE in the model that did not include cell type (5% FDR). **(A)** The median variance explained across genes in each cell type. (**B**) The effect size estimate in models with (*x-*axis) and without (*y-*axis) cell type proportion PCs.


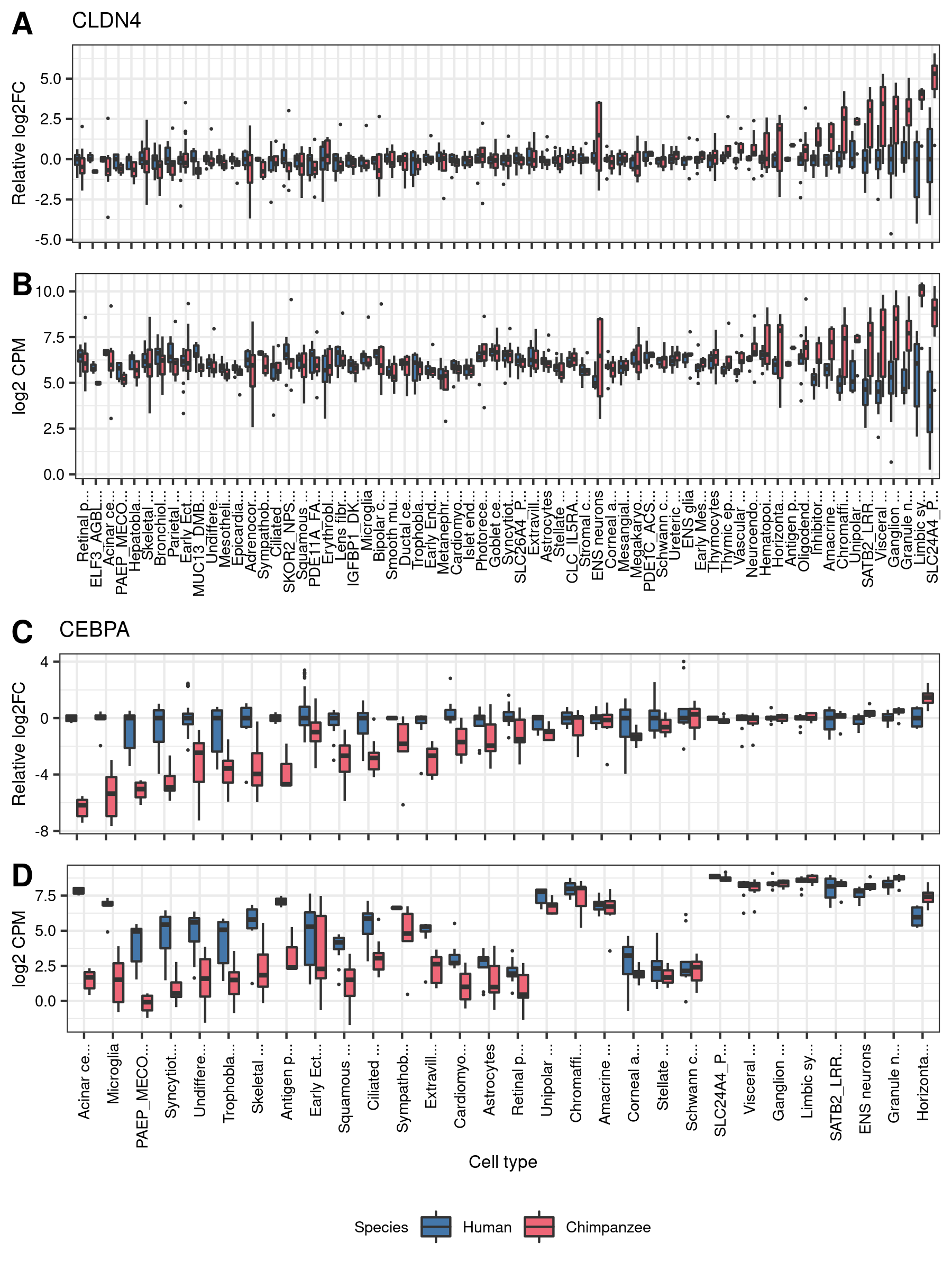


**Figure S10. Boxplots of *CLDN4* and *CEBPA* expression.** (**A**) Expression of *CLDN4* in all tested cell types, relative to the mean expression in humans. (**B**) Expression of *CLDN4* in all tested cell types. (**C**) Expression of *CEBPA* in all tested cell types, relative to the mean expression in humans. (**D**) Expression of *CEBPA* in all tested cell types.

**Tables S1 to S8**

|  | Replicate1 | Replicate2 | Replicate3 |
| --- | --- | --- | --- |
| NA19160 | 7921 | 4937 | 7186 |
| NA18511 | 5383 | 5150 | 6068 |
| NA18858 | 6805 | 5099 | 5385 |
| H28834 | 5801 |  |  |
| H21792 | 5851 |  |  |
| H28126 | 5962 |  |  |
| C40280 | 3737 | 3569 | 6841 |
| C3651 | 5586 | 4615 | 4652 |
| C3649 | 5263 | 3560 | 5898 |

**Table S1. Number of cells from each individual and replicate after quality control filters.**

| Cluster | Human | Chimpanzee |
| --- | --- | --- |
| 0 | 20921 | 24653 |
| 1 | 20501 | 398 |
| 2 | 4650 | 5931 |
| 3 | 7061 | 910 |
| 4 | 4106 | 2554 |
| 5 | 2119 | 3663 |
| 6 | 4702 | 379 |
| 7 | 3592 | 1196 |
| 8 | 653 | 2742 |
| 9 | 1704 | 1102 |
| 10 | 1539 | 193 |

**Table S2. Number of cells from each species in each cluster without reference integration.** Clusters are those defined by Seurat at resolution 0.1, and are illustrated in **Figure S2B**.

|  | Cell Type | Human | Chimpanzee | Hybrid |
| --- | --- | --- | --- | --- |
| 1 | Acinar cells | 100 | 161 | 86 |
| 2 | Adrenocortical cells | 155 | 143 | 59 |
| 3 | Amacrine cells | 237 | 135 | 794 |
| 4 | Antigen presenting cells | 35 | 19 | 19 |
| 5 | Astrocytes | 1430 | 859 | 8000 |
| 6 | Bipolar cells | 126 | 137 | 206 |
| 7 | Bronchiolar and alveolar epithelial cells | 187 | 443 | 168 |
| 8 | CLC_IL5RA positive cells | 69 | 23 | 19 |
| 9 | Cardiomyocytes | 844 | 174 | 995 |
| 10 | Chromaffin cells | 12 | 120 | 581 |
| 11 | Ciliated epithelial cells | 319 | 723 | 407 |
| 12 | Corneal and conjunctival epithelial cells | 339 | 84 | 385 |
| 13 | Ductal cells | 554 | 128 | 239 |
| 14 | ELF3_AGBL2 positive cells | 88 | 30 | 73 |
| 15 | ENS glia | 236 | 607 | 90 |
| 16 | ENS neurons | 642 | 400 | 229 |
| 17 | Early Ectoderm 1 | 14187 | 7751 | 7865 |
| 18 | Early Ectoderm 2 | 6876 | 6135 | 4491 |
| 19 | Early Ectoderm 3 | 643 | 469 | 176 |
| 20 | Early Ectoderm 4 | 87 | 18 | 132 |
| 21 | Early Endoderm 1 | 537 | 486 | 1256 |
| 22 | Early Mesoderm 1 | 755 | 82 | 441 |
| 23 | Epicardial fat cells | 464 | 144 | 572 |
| 24 | Erythroblasts | 127 | 132 | 52 |
| 25 | Extravillous trophoblasts | 125 | 76 | 12 |
| 26 | Ganglion cells | 229 | 361 | 358 |
| 27 | Goblet cells | 634 | 127 | 206 |
| 28 | Granule neurons | 212 | 208 | 729 |
| 29 | Hematopoietic stem cells | 165 | 55 | 132 |
| 30 | Hepatoblasts | 93 | 547 | 159 |
| 31 | Horizontal cells | 324 | 139 | 436 |
| 32 | IGFBP1_DKK1 positive cells | 1122 | 270 | 1237 |
| 33 | Inhibitory interneurons | 34 | 102 | 435 |
| 34 | Islet endocrine cells | 1083 | 1443 | 403 |
| 35 | Lens fibre cells | 53 | 411 | 89 |
| 36 | Limbic system neurons | 714 | 457 | 1928 |
| 37 | MUC13_DMBT1 positive cells | 164 | 168 | 63 |
| 38 | Megakaryocytes | 123 | 425 | 103 |
| 39 | Mesangial cells | 646 | 167 | 608 |
| 40 | Mesothelial cells | 195 | 148 | 149 |
| 41 | Metanephric cells | 527 | 91 | 216 |
| 42 | Microglia | 267 | 193 | 140 |
| 43 | Neuroendocrine cells | 163 | 217 | 159 |
| 44 | Oligodendrocytes | 182 | 201 | 1209 |
| 45 | PAEP_MECOM positive cells | 215 | 85 | 16 |
| 46 | PDE11A_FAM19A2 positive cells | 92 | 186 | 205 |
| 47 | PDE1C_ACSM3 positive cells | 53 | 31 | 74 |
| 48 | Parietal and chief cells | 185 | 192 | 29 |
| 49 | Photoreceptor cells | 176 | 158 | 91 |
| 50 | Retinal pigment cells | 707 | 668 | 1121 |
| 51 | Retinal progenitors and Muller glia | 2546 | 923 | 4464 |
| 52 | SATB2_LRRC7 positive cells | 80 | 288 | 736 |
| 53 | SKOR2_NPSR1 positive cells | 125 | 165 | 379 |
| 54 | SLC24A4_PEX5L positive cells | 263 | 305 | 1361 |
| 55 | SLC26A4_PAEP positive cells | 324 | 331 | 374 |
| 56 | Schwann cells | 3349 | 3538 | 792 |
| 57 | Skeletal muscle cells | 152 | 186 | 39 |
| 58 | Smooth muscle cells | 399 | 154 | 169 |
| 59 | Squamous epithelial cells | 523 | 342 | 1517 |
| 60 | Stellate cells | 526 | 34 | 397 |
| 61 | Stromal cells | 628 | 289 | 494 |
| 62 | Sympathoblasts | 20 | 114 | 19 |
| 63 | Syncytiotrophoblasts and villous cytotrophoblasts | 1255 | 328 | 358 |
| 64 | Thymic epithelial cells | 556 | 143 | 413 |
| 65 | Thymocytes | 307 | 200 | 189 |
| 66 | Trophoblast giant cells | 224 | 126 | 116 |
| 67 | Undifferentiated 1 | 13224 | 6442 | 4321 |
| 68 | Undifferentiated 2 | 6048 | 996 | 7166 |
| 69 | Unipolar brush cells | 108 | 144 | 112 |
| 70 | Ureteric bud cells | 107 | 284 | 296 |
| 71 | Vascular endothelial cells | 349 | 375 | 132 |
| 72 | Visceral neurons | 283 | 647 | 1115 |

**Table S3. Number of cells from each species assigned to each reference cell type.** Cell types are defined by the reference integration procedure described in Materials and Methods.

|  | Cell Type | DREAM | CorMotif | Hybrid |
| --- | --- | --- | --- | --- |
| 1 | Acinar cells | 1509 | 4764 | 228 |
| 2 | Adrenocortical cells | 346 | 4188 | 140 |
| 3 | Amacrine cells | 138 | 1104 | 2429 |
| 4 | Antigen presenting cells | 42 | 779 | 0 |
| 5 | Astrocytes | 3681 | 6640 | 8579 |
| 6 | Bipolar cells | 511 | 2768 | 1146 |
| 7 | Bronchiolar and alveolar epithelial cells | 879 | 3950 | 602 |
| 8 | CLC_IL5RA positive cells | 0 | 429 | 0 |
| 9 | Cardiomyocytes | 563 | 2062 | 7591 |
| 10 | Chromaffin cells | 2 | 27 | 1879 |
| 11 | Ciliated epithelial cells | 794 | 3224 | 1882 |
| 12 | Corneal and conjunctival epithelial cells | 91 | 604 | 4107 |
| 13 | Ductal cells | 888 | 1976 | 2618 |
| 14 | ELF3_AGBL2 positive cells | 7 | 410 | 1007 |
| 15 | ENS glia | 516 | 2251 | 276 |
| 16 | ENS neurons | 614 | 2280 | 1758 |
| 17 | Early Ectoderm 1 | 3712 | 6709 | 8766 |
| 18 | Early Ectoderm 2 | 2034 | 6193 | 8538 |
| 19 | Early Ectoderm 3 | 492 | 2470 | 571 |
| 20 | Early Ectoderm 4 | 0 | 413 | 712 |
| 21 | Early Endoderm 1 | 433 | 4791 | 5589 |
| 22 | Early Mesoderm 1 | 89 | 625 | 2887 |
| 23 | Epicardial fat cells | 515 | 2232 | 5355 |
| 24 | Erythroblasts | 656 | 4622 | 167 |
| 25 | Extravillous trophoblasts | 609 | 2950 | 0 |
| 26 | Ganglion cells | 381 | 1874 | 1877 |
| 27 | Goblet cells | 1532 | 3139 | 3922 |
| 28 | Granule neurons | 450 | 1618 | 3068 |
| 29 | Hematopoietic stem cells | 105 | 911 | 840 |
| 30 | Hepatoblasts | 1200 | 3911 | 3567 |
| 31 | Horizontal cells | 197 | 1506 | 1689 |
| 32 | IGFBP1_DKK1 positive cells | 278 | 1526 | 5750 |
| 33 | Inhibitory interneurons | 33 | 349 | 1629 |
| 34 | Islet endocrine cells | 2218 | 4293 | 2661 |
| 35 | Lens fibre cells | 463 | 2558 | 382 |
| 36 | Limbic system neurons | 753 | 2050 | 5241 |
| 37 | MUC13_DMBT1 positive cells | 1358 | 3324 | 1003 |
| 38 | Megakaryocytes | 2118 | 5403 | 322 |
| 39 | Mesangial cells | 114 | 1188 | 3420 |
| 40 | Mesothelial cells | 1452 | 3120 | 2098 |
| 41 | Metanephric cells | 105 | 801 | 1783 |
| 42 | Microglia | 2233 | 5124 | 528 |
| 43 | Neuroendocrine cells | 340 | 1760 | 732 |
| 44 | Oligodendrocytes | 493 | 2866 | 3872 |
| 45 | PAEP_MECOM positive cells | 569 | 3695 | 0 |
| 46 | PDE11A_FAM19A2 positive cells | 525 | 2536 | 511 |
| 47 | PDE1C_ACSM3 positive cells | 206 | 1679 | 283 |
| 48 | Parietal and chief cells | 540 | 4398 | 19 |
| 49 | Photoreceptor cells | 583 | 2596 | 145 |
| 50 | Retinal pigment cells | 1451 | 4251 | 5188 |
| 51 | Retinal progenitors and Muller glia | 3135 | 5794 | 8121 |
| 52 | SATB2_LRRC7 positive cells | 34 | 1119 | 2236 |
| 53 | SKOR2_NPSR1 positive cells | 463 | 2896 | 1648 |
| 54 | SLC24A4_PEX5L positive cells | 350 | 1566 | 4160 |
| 55 | SLC26A4_PAEP positive cells | 1638 | 4633 | 1896 |
| 56 | Schwann cells | 1449 | 3821 | 4453 |
| 57 | Skeletal muscle cells | 436 | 4718 | 79 |
| 58 | Smooth muscle cells | 367 | 2484 | 1520 |
| 59 | Squamous epithelial cells | 476 | 3088 | 8054 |
| 60 | Stellate cells | 323 | 1490 | 4801 |
| 61 | Stromal cells | 799 | 3424 | 4015 |
| 62 | Sympathoblasts | 522 | 4216 | 0 |
| 63 | Syncytiotrophoblasts and villous cytotrophoblasts | 3019 | 6359 | 2167 |
| 64 | Thymic epithelial cells | 353 | 1860 | 5260 |
| 65 | Thymocytes | 1282 | 5115 | 738 |
| 66 | Trophoblast giant cells | 788 | 4134 | 455 |
| 67 | Undifferentiated 1 | 1682 | 7095 | 8993 |
| 68 | Undifferentiated 2 | 2073 | 5320 | 11471 |
| 69 | Unipolar brush cells | 292 | 1103 | 426 |
| 70 | Ureteric bud cells | 488 | 2667 | 1633 |
| 71 | Vascular endothelial cells | 993 | 3410 | 1400 |
| 72 | Visceral neurons | 160 | 1726 | 3228 |

**Table S4. Number of differentially expressed genes by cell type.** The number of differentially expressed cells calculated with DREAM at 5% false discovery rate, Cormotif and 95% posterior probability of DE, or in tetraploid hybrid cells using the Wilcoxon signed rank test at 5% false discovery rate.

| cormotif | GO.ID | Term | p-value |
| --- | --- | --- | --- |
| 1 | GO:0046474 | glycerophospholipid biosynthetic process | 0.0002 |
| 1 | GO:0016973 | poly(A)+ mRNA export from nucleus | 0.00031 |
| 1 | GO:0006357 | regulation of transcription by RNA polym... | 0.00073 |
| 1 | GO:0015012 | heparan sulfate proteoglycan biosyntheti... | 0.00077 |
| 1 | GO:0071108 | protein K48-linked deubiquitination | 0.00082 |
| 1 | GO:0016567 | protein ubiquitination | 0.00091 |
| 1 | GO:0016575 | histone deacetylation | 0.00116 |
| 1 | GO:0043967 | histone H4 acetylation | 0.0012 |
| 1 | GO:0031398 | positive regulation of protein ubiquitin... | 0.00204 |
| 1 | GO:0043968 | histone H2A acetylation | 0.00294 |
| 2 | GO:0007411 | axon guidance | 7.40E-05 |
| 2 | GO:0009880 | embryonic pattern specification | 0.00046 |
| 2 | GO:0045665 | negative regulation of neuron differenti... | 0.00083 |
| 2 | GO:0035019 | somatic stem cell population maintenance | 0.00099 |
| 2 | GO:0030857 | negative regulation of epithelial cell d... | 0.00104 |
| 2 | GO:0001822 | kidney development | 0.00117 |
| 2 | GO:0060037 | pharyngeal system development | 0.00153 |
| 2 | GO:0001657 | ureteric bud development | 0.00175 |
| 2 | GO:0014059 | regulation of dopamine secretion | 0.00205 |
| 2 | GO:0008360 | regulation of cell shape | 0.00257 |
| 3 | GO:0006357 | regulation of transcription by RNA polym... | 0.00083 |
| 3 | GO:0006468 | protein phosphorylation | 0.00178 |
| 3 | GO:0008643 | carbohydrate transport | 0.0028 |
| 3 | GO:0090110 | COPII-coated vesicle cargo loading | 0.00339 |
| 3 | GO:0015031 | protein transport | 0.00418 |
| 3 | GO:0070932 | histone H3 deacetylation | 0.00551 |
| 3 | GO:0006353 | DNA-templated transcription, termination | 0.00551 |
| 3 | GO:0031145 | anaphase-promoting complex-dependent cat... | 0.00551 |
| 3 | GO:0006066 | alcohol metabolic process | 0.00569 |
| 3 | GO:0043242 | negative regulation of protein-containin... | 0.00577 |
| 4 | GO:0006400 | tRNA modification | 0.00027 |
| 4 | GO:0042273 | ribosomal large subunit biogenesis | 0.00073 |
| 4 | GO:0030488 | tRNA methylation | 0.00145 |
| 4 | GO:0009130 | pyrimidine nucleoside monophosphate bios... | 0.00207 |
| 4 | GO:0006221 | pyrimidine nucleotide biosynthetic proce... | 0.00241 |
| 4 | GO:0034982 | mitochondrial protein processing | 0.00275 |
| 4 | GO:1902108 | regulation of mitochondrial membrane per... | 0.0049 |
| 4 | GO:0000463 | maturation of LSU-rRNA from tricistronic... | 0.00558 |
| 4 | GO:0009265 | 2'-deoxyribonucleotide biosynthetic proc... | 0.00558 |
| 4 | GO:0006364 | rRNA processing | 0.00593 |
| 5 | GO:0051301 | cell division | 5.80E-06 |
| 5 | GO:0010837 | regulation of keratinocyte proliferation | 0.00021 |
| 5 | GO:0006260 | DNA replication | 0.00064 |
| 5 | GO:0009185 | ribonucleoside diphosphate metabolic pro... | 0.00067 |
| 5 | GO:0000281 | mitotic cytokinesis | 0.00084 |
| 5 | GO:0051255 | spindle midzone assembly | 0.00122 |
| 5 | GO:0006222 | UMP biosynthetic process | 0.00122 |
| 5 | GO:0070316 | regulation of G0 to G1 transition | 0.00122 |
| 5 | GO:1900087 | positive regulation of G1/S transition o... | 0.00134 |
| 5 | GO:0070374 | positive regulation of ERK1 and ERK2 cas... | 0.00139 |
| 6 | GO:0050686 | negative regulation of mRNA processing | 0.00058 |
| 6 | GO:0090085 | regulation of protein deubiquitination | 0.00147 |
| 6 | GO:0032436 | positive regulation of proteasomal ubiqu... | 0.00201 |
| 6 | GO:0033120 | positive regulation of RNA splicing | 0.00232 |
| 6 | GO:0006974 | cellular response to DNA damage stimulus | 0.0032 |
| 6 | GO:0070536 | protein K63-linked deubiquitination | 0.00347 |
| 6 | GO:2001169 | regulation of ATP biosynthetic process | 0.00353 |
| 6 | GO:0006903 | vesicle targeting | 0.0042 |
| 6 | GO:1900363 | regulation of mRNA polyadenylation | 0.005 |
| 6 | GO:2000144 | positive regulation of DNA-templated tra... | 0.00549 |
| 7 | GO:1903543 | positive regulation of exosomal secretio... | 2.60E-05 |
| 7 | GO:0070584 | mitochondrion morphogenesis | 0.00027 |
| 7 | GO:0042246 | tissue regeneration | 0.00061 |
| 7 | GO:0099171 | presynaptic modulation of chemical synap... | 0.00089 |
| 7 | GO:0008333 | endosome to lysosome transport | 0.00116 |
| 7 | GO:0010889 | regulation of sequestering of triglyceri... | 0.00135 |
| 7 | GO:0051561 | positive regulation of mitochondrial cal... | 0.00135 |
| 7 | GO:0045736 | negative regulation of cyclin-dependent ... | 0.00186 |
| 7 | GO:0060211 | regulation of nuclear-transcribed mRNA p... | 0.00231 |
| 7 | GO:0007049 | cell cycle | 0.00301 |
| 8 | GO:0007411 | axon guidance | 4.30E-06 |
| 8 | GO:0001525 | angiogenesis | 5.70E-05 |
| 8 | GO:0045669 | positive regulation of osteoblast differ... | 6.50E-05 |
| 8 | GO:0001764 | neuron migration | 0.00015 |
| 8 | GO:0009952 | anterior/posterior pattern specification | 0.00023 |
| 8 | GO:0072112 | glomerular visceral epithelial cell diff... | 0.00025 |
| 8 | GO:0060021 | roof of mouth development | 0.00031 |
| 8 | GO:0060841 | venous blood vessel development | 0.00038 |
| 8 | GO:0021846 | cell proliferation in forebrain | 0.00043 |
| 8 | GO:0097094 | craniofacial suture morphogenesis | 0.00053 |
| 9 | GO:2000105 | positive regulation of DNA-templated DNA... | 8.70E-05 |
| 9 | GO:0023019 | signal transduction involved in regulati... | 0.00056 |
| 9 | GO:0106074 | aminoacyl-tRNA metabolism involved in tr... | 0.00063 |
| 9 | GO:0021517 | ventral spinal cord development | 0.00142 |
| 9 | GO:0022406 | membrane docking | 0.00326 |
| 9 | GO:0036508 | protein alpha-1,2-demannosylation | 0.00371 |
| 9 | GO:0050919 | negative chemotaxis | 0.00398 |
| 9 | GO:0051095 | regulation of helicase activity | 0.00449 |
| 9 | GO:0010454 | negative regulation of cell fate commitm... | 0.00449 |
| 9 | GO:1990748 | cellular detoxification | 0.00585 |
| 10 | GO:0008360 | regulation of cell shape | 2.80E-06 |
| 10 | GO:0030335 | positive regulation of cell migration | 8.70E-06 |
| 10 | GO:0051900 | regulation of mitochondrial depolarizati... | 9.70E-06 |
| 10 | GO:0055010 | ventricular cardiac muscle tissue morpho... | 4.40E-05 |
| 10 | GO:0007229 | integrin-mediated signaling pathway | 0.00018 |
| 10 | GO:0048251 | elastic fiber assembly | 0.00025 |
| 10 | GO:0010811 | positive regulation of cell-substrate ad... | 0.00025 |
| 10 | GO:0050731 | positive regulation of peptidyl-tyrosine... | 0.00059 |
| 10 | GO:0008347 | glial cell migration | 0.0006 |
| 10 | GO:0045669 | positive regulation of osteoblast differ... | 0.00099 |
| 11 | GO:0061844 | antimicrobial humoral immune response me... | 2.00E-06 |
| 11 | GO:0007586 | digestion | 5.20E-06 |
| 11 | GO:0019731 | antibacterial humoral response | 9.90E-06 |
| 11 | GO:0035082 | axoneme assembly | 1.40E-05 |
| 11 | GO:0003341 | cilium movement | 1.80E-05 |
| 11 | GO:0007218 | neuropeptide signaling pathway | 1.90E-05 |
| 11 | GO:0007339 | binding of sperm to zona pellucida | 6.30E-05 |
| 11 | GO:0007288 | sperm axoneme assembly | 9.20E-05 |
| 11 | GO:0030858 | positive regulation of epithelial cell d... | 0.00011 |
| 11 | GO:0050829 | defense response to Gram-negative bacter... | 0.00015 |
| 12 | GO:1900112 | regulation of histone H3-K9 trimethylati... | 0.00012 |
| 12 | GO:0060261 | positive regulation of transcription ini... | 0.00082 |
| 12 | GO:0006449 | regulation of translational termination | 0.00135 |
| 12 | GO:0051573 | negative regulation of histone H3-K9 met... | 0.00135 |
| 12 | GO:0033169 | histone H3-K9 demethylation | 0.00216 |
| 12 | GO:0000462 | maturation of SSU-rRNA from tricistronic... | 0.00218 |
| 12 | GO:0007264 | small GTPase mediated signal transductio... | 0.0024 |
| 12 | GO:0006448 | regulation of translational elongation | 0.0028 |
| 12 | GO:0042058 | regulation of epidermal growth factor re... | 0.00281 |
| 12 | GO:0097502 | mannosylation | 0.00327 |
| 13 | GO:0035924 | cellular response to vascular endothelia... | 0.00014 |
| 13 | GO:0002181 | cytoplasmic translation | 0.00024 |
| 13 | GO:0097084 | vascular associated smooth muscle cell d... | 0.00027 |
| 13 | GO:1904667 | negative regulation of ubiquitin protein... | 0.0004 |
| 13 | GO:0046530 | photoreceptor cell differentiation | 0.00293 |
| 13 | GO:0070918 | primary sncRNA processing | 0.00294 |
| 13 | GO:0003183 | mitral valve morphogenesis | 0.00321 |
| 13 | GO:0009642 | response to light intensity | 0.00321 |
| 13 | GO:0097104 | postsynaptic membrane assembly | 0.00321 |
| 13 | GO:0048485 | sympathetic nervous system development | 0.00388 |

**Table S5. Gene Ontology enrichment for cormotifs.** We assigned each gene to the cormotif for which it had the greatest posterior probability of membership, then ran GO enrichment for biological process. The top 10 terms for each cormotif are presented.

|  | GO.ID | Term | p-value |
| --- | --- | --- | --- |
| 1 | GO:0008380 | RNA splicing | 0.00042 |
| 2 | GO:0045931 | positive regulation of mitotic cell cycl... | 0.00044 |
| 3 | GO:0006397 | mRNA processing | 0.00049 |
| 4 | GO:0032007 | negative regulation of TOR signaling | 0.00197 |
| 5 | GO:0006513 | protein monoubiquitination | 0.00212 |
| 6 | GO:0043966 | histone H3 acetylation | 0.00294 |
| 7 | GO:0051098 | regulation of binding | 0.00318 |
| 8 | GO:0016236 | macroautophagy | 0.00392 |
| 9 | GO:0016032 | viral process | 0.00433 |
| 10 | GO:0000278 | mitotic cell cycle | 0.00515 |
| 11 | GO:0016925 | protein sumoylation | 0.00552 |
| 12 | GO:0032663 | regulation of interleukin-2 production | 0.00832 |
| 13 | GO:0051028 | mRNA transport | 0.00951 |
| 14 | GO:0006367 | transcription initiation from RNA polyme... | 0.01289 |
| 15 | GO:0043161 | proteasome-mediated ubiquitin-dependent ... | 0.01318 |
| 16 | GO:0000338 | protein deneddylation | 0.01594 |
| 17 | GO:2000757 | negative regulation of peptidyl-lysine a... | 0.01594 |
| 18 | GO:0043967 | histone H4 acetylation | 0.01598 |
| 19 | GO:0006606 | protein import into nucleus | 0.0161 |
| 20 | GO:0016567 | protein ubiquitination | 0.01703 |

**Table S6. Gene Ontology enrichment in genes not differentially expressed in any cell type.** The gene set was filtered for genes that had at least 100 UMIs detected across all individuals and replicates in at least 10 cell types. The top 20 terms are provided.

|  | pathway | Adjusted  p-value | Normalized enrichment score |
| --- | --- | --- | --- |
| 1 | TUBE_MORPHOGENESIS | 9.80E-10 | 1.8 |
| 2 | TUBE_DEVELOPMENT | 2.20E-09 | 1.8 |
| 3 | REGULATION_OF_MULTICELLULAR_ ORGANISMAL_DEVELOPMENT | 9.80E-09 | 1.7 |
| 4 | CELL_FATE_COMMITMENT | 1.10E-08 | 2.3 |
| 5 | TISSUE_DEVELOPMENT | 2.90E-08 | 1.6 |
| 6 | ANIMAL_ORGAN_MORPHOGENESIS | 4.20E-08 | 1.8 |
| 7 | ANATOMICAL_STRUCTURE_ FORMATION_INVOLVED_IN_MORP... | 1.30E-07 | 1.7 |
| 8 | EMBRYO_DEVELOPMENT | 2.20E-07 | 1.7 |
| 9 | ENZYME_LINKED_RECEPTOR_ PROTEIN_SIGNALING_PATHWAY | 2.20E-07 | 1.7 |
| 10 | BIOLOGICAL_ADHESION | 2.40E-07 | 1.6 |
| 11 | REGULATION_OF_CELL_POPULATION_ PROLIFERATION | 6.00E-07 | 1.6 |
| 12 | EMBRYONIC_ORGAN_DEVELOPMENT | 6.50E-07 | 1.9 |
| 13 | CELL_CYCLE | 1.40E-06 | 1.5 |
| 14 | EMBRYONIC_MORPHOGENESIS | 2.00E-06 | 1.8 |
| 15 | CIRCULATORY_SYSTEM_ DEVELOPMENT | 2.50E-06 | 1.6 |
| 16 | EPITHELIUM_DEVELOPMENT | 6.10E-06 | 1.6 |
| 17 | REGULATION_OF_CELL_ DIFFERENTIATION | 6.10E-06 | 1.5 |
| 18 | PATTERN_SPECIFICATION_PROCESS | 1.20E-05 | 1.9 |
| 19 | POSITIVE_REGULATION_OF_CELL_ POPULATION_PROLIFER... | 1.30E-05 | 1.7 |
| 20 | POSITIVE_REGULATION_OF_ DEVELOPMENTAL_PROCESS | 1.50E-05 | 1.5 |

**Table S7. Gene Ontology enrichment for *trans* genes.** We ranked genes according to the mean *trans* proportion across all cell types with DE and ran gene set enrichment against Gene Ontology Biological Process terms. The top 20 terms are provided.

|  | Pathway | Adjusted  p-value | Normalized enrichment score |
| --- | --- | --- | --- |
| 1 | TFT_LEGACY_TAATTA_CHX10_01 | 2.90E-06 | 1.74 |
| 2 | TFT_LEGACY_CTTTGA_LEF1_Q2 | 5.50E-06 | 1.62 |
| 3 | TFT_GTRD_HSD17B8_TARGET_GENES | 5.00E-05 | 1.69 |
| 4 | MIR_DB_MIR144_3P | 1.90E-04 | 1.75 |
| 5 | MIR_DB_MIR8485 | 5.80E-04 | 1.5 |
| 6 | TFT_LEGACY_GGATTA_PITX2_Q2 | 5.80E-04 | 1.64 |
| 7 | TFT_LEGACY_YCATTAA_UNKNOWN | 5.80E-04 | 1.69 |
| 8 | MIR_DB_MIR9_5P | 6.50E-04 | 1.68 |
| 9 | TFT_LEGACY_CEBPDELTA_Q6 | 1.10E-03 | 1.88 |
| 10 | TFT_LEGACY_CEBPA_01 | 1.30E-03 | 1.86 |
| 11 | TFT_LEGACY_HNF1_C | 1.30E-03 | 1.9 |
| 12 | TFT_LEGACY_CAGCTG_AP4_Q5 | 1.30E-03 | 1.43 |
| 13 | TFT_LEGACY_AACTTT_UNKNOWN | 1.30E-03 | 1.39 |
| 14 | TFT_LEGACY_GGGTGGRR_PAX4_03 | 1.70E-03 | 1.42 |
| 15 | TFT_LEGACY_WGGAATGY_TEF1_Q6 | 2.30E-03 | 1.72 |
| 16 | TFT_LEGACY_RYTTCCTG_ETS2_B | 2.60E-03 | 1.47 |
| 17 | TFT_LEGACY_GATA1_05 | 3.00E-03 | 1.77 |
| 18 | TFT_LEGACY_IRF7_01 | 3.10E-03 | 1.81 |
| 19 | TFT_LEGACY_CAGGTA_AREB6_01 | 3.20E-03 | 1.52 |
| 20 | MIR_DB_MIR4699_3P | 3.20E-03 | 1.58 |

**Table S8. Transcription factor target enrichment for *trans* genes.** We ranked genes according to the mean *trans* proportion across all cell types with DE and ran gene set enrichment against transcription factor targets. The top 20 terms are provided.
