# Supplementary material for: The relationship between regulatory changes in cis and trans and the evolution of gene expression in humans and chimpanzees
Source: Genome Biol. 2023 Sep 11;24:207. doi: 10.1186/s13059-023-03019-3 (PMC10496171; doi:10.1186/s13059-023-03019-3)
Supplement: Supplementary file 2 — Additional file 2. Data S1. A csv file with summary statistics for all DE tests and calculated cis-proportion. Columns are described in Additional file 1. [file 13059_2023_3019_MOESM2_ESM.zip › Data S1.docx]

**Data S1. Results of DREAM, Cormotif, Hybrid DE, and cis proportion.** This supplementary data file is a flat tab-separated text file with the following columns defined:

gene The gene symbol for the tested gene.

cell.type The reference cell type the gene was tested in (see materials and methods).

layer The inferred germ layer of this cell type.

numi.human The number of UMIs detected across all human individuals and replicates.

numi.chimp The number of UMIs detected across all chimpanzee individuals and replicates.

dream.mean.human Mean gene expression across humans, calculated by DREAM. UMI counts are log2 transformed as well as cyclicloess and TMM normalized.

dream.mean.chimp Mean gene expression across chimpanzees, calculated by DREAM. UMI counts are log2 transformed as well as cyclicloess and TMM normalized.

dream.logFC The log2 fold-change (the effect size) in gene expression in humans relative to chimpanzees, calculated by DREAM.

dream.SE The standard error in the DREAM effect size estimate.

dream.mean The mean expression of this gene across both humans and chimpanzees, as calculated by DREAM. UMI counts are log2 transformed as well as cyclicloess and TMM normalized.

dream.p.val The raw p-value computed by DREAM.

dream.adj.pval The Benjamini-Hochberg adjusted p-value, computed by DREAM.

dream.p.conserved The probability that the absolute logFC is less than 0.5

cormotif.posterior The posterior probability of differential expression, computed by Cormotif.

cormotif.cluster The correlation motif for which this gene has the highest posterior probability of membership.

hybrid.numi.human The number of human-assigned UMIs detected across all replicates of tetraploid human/chimpanzee hybrid cells.

hybrid.numi.chimp The number of chimpanzee-assigned UMIs detected across all replicates of tetraploid human/chimpanzee hybrid cells.

hybrid.logFC The hybrid effect size. Computed as the difference between log2(hybrid.numi.human + 1) and log2(hybrid.numi.human + 1). The pseudocount is included to prevent taking the logarithm of zero.

hybrid.p.val The p-value of the difference between human and chimpanzee assigned counts, computed using a paired Wilcoxon signed-rank test .

hybrid.adj.pval The Benjamini-Hochberg adjusted p-value in for the hybrid test.

cisprop The estimated *cis* proportion of this change. Calculated as abs(hybrid.logFC) / ( abs(dream.logFC – hybrid.logFC) + abs(hybrid.logFC) )
