# Supplementary material for: The relationship between regulatory changes in cis and trans and the evolution of gene expression in humans and chimpanzees
Source: Genome Biol. 2023 Sep 11;24:207. doi: 10.1186/s13059-023-03019-3 (PMC10496171; doi:10.1186/s13059-023-03019-3)
Supplement: Supplementary file 3 — Additional file 3. Data S2. A Raw text file containing the gene symbol of each gene with cell-type-restricted differential expression. One gene is listed per line. [file 13059_2023_3019_MOESM3_ESM.zip › Data S2.docx]

**Data S2. Genes with cell-type restricted differential expression.** Raw text file containing the gene symbol of each gene with cell-type-restricted differential expression. One gene is listed per line.
