# Supplementary material for: The relationship between regulatory changes in cis and trans and the evolution of gene expression in humans and chimpanzees
Source: Genome Biol. 2023 Sep 11;24:207. doi: 10.1186/s13059-023-03019-3 (PMC10496171; doi:10.1186/s13059-023-03019-3)
Supplement: Supplementary file 4 — Additional file 4. Data S3. Statistics for tests of enrichment in TFs in promoters, and correlated DE between TFs and putative target genes. Columns are described in Additional file 1. [file 13059_2023_3019_MOESM4_ESM.zip › Data S3.docx]

**Data S3. Transcription factors, putative target genes, and correlated patterns of DE.** Flat comma-separated text file with the following columns:

TF The gene symbol for the transcription factor.

motif The most significantly enriched motif in the msigDB TF legacy set that is associated with this TF.

hypergeom.adj.pval The FDR-adjusted p-value that this motif is enriched in the promoters of *trans*-DE genes relative to all tested genes

combined.adj.pval The FDR-adjusted Fishers combined p-value testing for excess enrichment in correlated DE patters of TFs and their putative target genes.

target A gene containing the motif of the TF in column 1 in its promoter.

cor The Pearson correlation in the DE pattern across cell types of the TF and the target

cor.pval The p-value of this correlation

cor.adj.pval The FDR-adjusted p-value of this correlation

**Seurat object for interactive data exploration.**

We have included a Seurat object for easy access to the data. This file is available under GEO assesion GSE201516. This h5Seurat file can be imported into R/Rstudio or converted to an Anndata object for use in Python using the SeuratData package in R.

The file contains both diploid and tetraploid datasets embedded within the same UMAP space. The combined human/chimpanzee genome alignment can only assign reads that uniquely map to a single species. For that reason it is difficult to directly compare mean levels of gene expression between diploid and tetraploid analysis. For that reason, we included tetraploid data aligned to the human genome alone in addition to the combined genome. For the combined genome alignments we have split the reads that align to each species into a separate pseudocell.
