# Supplementary material for: The relationship between regulatory changes in cis and trans and the evolution of gene expression in humans and chimpanzees
Source: Genome Biol. 2023 Sep 11;24:207. doi: 10.1186/s13059-023-03019-3 (PMC10496171; doi:10.1186/s13059-023-03019-3)
Supplement: Supplementary file 5 — Additional file 5. Peer review history. [file 13059_2023_3019_MOESM5_ESM.docx]

**Review history**

**First round of review**

**Reviewer 1**

Barr et al present a large collection of scRNA data derived from human and chimpanzee embryoid bodies and the many cell types they contain. They focus their analyses on inter-species differences in expression and, by extension, regulation. The analyses are complemented by the inclusion of a second large dataset of EBs from a fused human/chimpanzee tetraploid line, which allows them to begin separating cis vs trans contribution to interspecies differences in gene expression.

The resulting dataset is framed as a resource for comparative genomics studies, and as a transformative advancement over the state of the field in terms of human/chimpanzee gene expression data sets. In that sense, the manuscript is successful - a lot of questions about the nature of gene expression and regulation and their role in evolution follow naturally from it. The authors do a good job at teasing some of these out. However, while EBs are a big advancement on what came before, they are still an imperfect model. To establish them as a new paradigm I think the analyses need to delve a bit further, although I accept this decision is ultimately up to the authors.

It would also be great if this data were deposited in a somewhat processed form somewhere for others to query interactively (at the individual cell level), but I recognize that's a stretch goal. I also couldn't find any guidance on raw data access beyond the three supplementary tables, which should be addressed.

Major comments:
DE results:
The authors say that there's a surprising number of genes that are not DE (cormotif analyses, discussion), which they attribute in part to incomplete power. Do the authors have an idea of how incomplete their power actually is? I realize this is a hard thing to test so I leave the means - and extent - of addressing it open. But for example, if the authors had data from 10 different chimps would every gene be DE somewhere? Do we expect this under evolutionary models of gene regulation and species divergence, or is there a point to be made here about statistical and biological importance? The paper does not mention the distribution of log FC magnitudes at any point; what does this curve look like? Different by tissue? I realize LOEUF, dN/dS etc are circling around this point too, so my suggestion could be integrated with those analyses.

Cormotif and tissue specificity:
Do the patterns of tissue specificity make sense, evolutionarily speaking? Some of the clusters in 2A are 'nice' biologically, in that they seem to impact similar related cells (eg 7, 13), but others seem noisier and messier. Mesendoderm and ectoderm mostly separate from each other, but not fully. In addition, is there anything to be gained by comparing the results here with DE result obtained from previous studies of chimpanzee/human differences in post mortem tissue?


Cell numbers and variability:
Although the authors repeatedly mention that cell numbers are similar/roughly comparable/consistent across species, I don't always agree, and these claims are never properly tested. In Table S1 cell numbers seem consistently sparser for chimps than humans, in Table S2/pg 7 line 19 6/11 clusters have fairly different cell numbers, and in Table S3, pg 7 line 46 there's clear differences in abundance in some cell types, in particular the abundance of undifferentiated cells. Is there a role for differences in make up/cell crosstalk in driving observed DE differences? I would find their claims more reassuring if they were backed up.


Minor comments:
Pg 6, line 43: "We also included a single replicate of EBs from three additional human lines of European ancestry to confirm that inter-species differences are not population-specific." Where are the results of this analysis?

pg 8, line 44 onwards: References to Fig 2 panels are incorrect through this section, eg pg 8, line 56 and pg 9, line 4.

pg 15, line 53: The Barreiro paper doesn't test that chimpanzee cells resist progression from HIV to AIDS. Is there a citation missing here?

pg 19, line 14: There's a lot of detail here on the sequencing and collection set up, but I actually have no idea of what replicates and lanes are associated with what library? Is this info somewhere else? If so, a call out here would be nice.

pg 20, line 11: "Finally, we used BLAT to the map the" should be "to map the"

pg 21, line 37: "a substantial number of cells" how many?

pg 24, line 55: "LOEUF was obtained from a study of 141,456 human genomes (Karczewski et al., 2020)." Genomes should be 'exomes'; gnomAD v2 contains 125,748 exomes and 15,708 genomes, v3 ~75k genomes.

pg 25, line 39: "We manually annotated this set of 179 motifs to identify the TF that binds to each motif." How? (One sentence is probably enough explanation here)

pg 58, line 58: "The inferred germ later of this cell type." later should be layer.

**Reviewer 2**

Barr, Rhodes and Gilad performed single cell transcriptomic analysis of embryoid bodies derived from human, chimp and tetraploid human-chimp iPSC lines. Authors use this system to obtain insights into gene expression differences between human and chimpanzee cells during early cell fate decisions. First, they analyze transcriptomic data from diploid human and chimp embryoid bodies. They detect 86 distinct cell clusters, most matching previously known cell types. Next, the authors employ two approaches in pseudo-bulked data to detect gene expression differences that can be explained by the species of origin: a) within cluster differential analysis b) a cormotif approach, which is a more global approach that fits a Bayesian hierarchical model to find a set of genes that might be correlated between samples. As expected, the second approach has more statistical power but both approaches find an ample amount of differentially expressed genes. The two predominant expression 'motifs' consists of genes that either show differential expression in all cell clusters or in none. Remaining motifs show more nuanced differential expression restricted only to certain cell types.

In the second part of the manuscript, the authors apply single cell technology to analyze differential expression in embryoid bodies derived from hybrid human-chimp tetraploid lines. This system has an advantage by providing a common "trans" environment that allowing for robust detection of cis-effects i.e. those explainable DNA sequence differences in a given gene regulatory environment. Indeed authors find that most of DE detected in single species parent lines are attributable to the "trans" effects. Based on the comparisons of the species-specific expression differences in hybrid vs diploid cells, the authors infer which changes are due to trans vs cis-effects. Subsequent analysis of differentially expressed transcription factors and their motifs suggests candidate mediators of these effects.

Overall, this is an interesting manuscript and a valuable resource for the community, and as such, appropriate for publication in Genome Biology. However, the study suffers from some confounding effects and over-interpretations, which should at least be acknowledged in the revised manuscript.

Major comments:

1. Authors claim that genes showing differential expression in cis to have more divergent sequences and possibly relaxed evolutionary constraints. However there exist a possibility that the more divergent genes are more likely to experience some systematic alignment bias that may skew the apparent relative expression difference between species, particularly considering that samples may have considerable sequence polymorphisms relative to reference genomes and that about 5% of chimpanzee genome sequence might not yet be accounted for, but might be generating reads. Authors should perform alignments with simulated reads of various quality to determine to what extent this can create systematic DE skew. Also reporting explicitly distribution of fold-changes would be helpful interpreting these results - how large are effects? The z-scores read from heatmap appear to be contained within (-1.5,1.5) range so not very large. With powerful statistical analysis comes risk of detection of smaller and possibly unrelated effects such as subtle alignment bias.

2. A major source of strong correlated DE between most cell types could come from uneven differentiation potential of the cell lines - either inherently due to the species differences or due to technical issues with tissue culture that has not been optimized for the chimp cells. Such possibility is suggested by a latter section of the manuscript where authors analyze tetraploid lines which are internally controlled against such effects (and then claim massive trans-environment effects (fig 3B)) and also by quite dramatic differences in relative cell type frequencies between species, which are somewhat unexpected (fig 1D vs 1E, and the supplementary tables). Indeed, a trans effect that would create such correlated changes across cell types would likely originate from a shared stimulus, such as a metabolic effect. I may have missed it but what are the GO categories of genes in motif 11 (shared DE effect).

The authors swept multiple potential confounding sources of DE into one label of "trans". This needs to be explicitly acknowledged in the paper and ideally, an attempt at separating these different sources of variation should be made. There are at least three sources of trans-effects here: 1) trans-regulatory environment - differences in sequences and molar concentration of proteins within cells of different species; 2) observed differences in frequencies of cell types within embryoid bodies that create a different signaling and metabolic environments, which affect expression in cell-autonomous and non-autonomous ways; 3) possibly suboptimal culture conditions for at least one species (which may also may be related to the second source of variation); in addition there might be additional effects due to derivation and epigenetic differences between the lines. In my opinion authors should try to disentangle some of these effects, and in the absence of it, authors should properly discuss the nuances of the "trans" effects and limitations of the analysis in these respect. In particular, the type-2 trans effects can be accessible if the individual embryoid bodies are identifiable in sequencing (though depending on the technicalities that information could have been lost). It is also not clear to me why the tetraploid set was analyzed separately and seemingly with somewhat different methodology. Surely combining the data sets might provide a stronger more robust analysis.

Minor points:
The use of "motif" for cormotif gene sets is unfortunate - I know it comes from the package terminology - but it might be confused with sequence motifs, which are also considered in the manuscript.

The manuscript text/figures requires some polishing - there appears to be discrepancies between text and figures, for instance panels 2E and 2F are switched in the text relative to figure, with somewhat different p value given.

**Authors’ response to reviewers**

Reviewer Responses

We thank the reviewers for their helpful comments. Below, we provide point-by-point responses to each reviewer. Changes to the manuscript text are highlighted. We have also added five new supplementary figures (S3-S6, S9) and one new supplementary table (S5) in response to reviewer suggestions.

# Reviewer 1

*Description Comment 1*

In their description of the work, reviewer 1 states:

*It would also be great if this data were deposited in a somewhat processed form somewhere for others to query interactively (at the individual cell level), but I recognize that's a stretch goal.*

We agree that it would be wonderful to have the data in a place where it could be queried interactively. We looked for public repositories that could be a good fit for this data; however, we were unable to find one that would facilitate easy comparison between the human and chimpanzee datasets.

Still, we would like users to be able to use our data interactively. To address this, we added an h5Seurat file to the GEO repository, which is publicly available at GSE201516. This will allow users to easily obtain the data and interact with it using R/Rstudio and Seurat. A description of this file has been added to the supplementary materials under Data S4.

*Description Comment 2*

In their description, reviewer 1 also states:

*I also couldn't find any guidance on raw data access beyond the three supplementary tables, which should be addressed.*

All the raw and processed data files and their descriptions are available through GEO. Previously, this was restricted access, however we have now made the GEO repository public. It can be accessed at GSE201516.

*Major Comment 1*

In their first major comment, reviewer 1 states:

*The authors say that there's a surprising number of genes that are not DE (cormotif analyses, discussion), which they attribute in part to incomplete power. Do the authors have an idea of how incomplete their power actually is? I realize this is a hard thing to test so I leave the means - and extent - of addressing it open. But for example, if the authors had data from 10 different chimps would every gene be DE somewhere? Do we expect this under evolutionary models of gene regulation and species divergence, or is there a point*

*to be made here about statistical and biological importance? The paper does not mention the distribution of log FC magnitudes at any point; what does this curve look like? Different by tissue? I realize LOEUF, dN/dS etc are circling around this point too, so my suggestion could be integrated with those analyses.*

There are a number of questions to address in the comment. We will address each separately.

*How incomplete is power?*

We attempted to control for power and enrich for truly non-DE genes by applying UMI count thresholds. We argued that at large UMI count thresholds it is extremely unlikely that power can explain the lack of DE. In other words, we would not expect to find DE in these genes even with a large number of individuals.

We recognize that this makes it difficult to quantify how confident we are that any particular gene is not DE. To address this we have added a new analysis. Using summary statistics from our linear mixed model, we calculated the probability that each absolute effect size was smaller than 0.5. We found that at a 95% confidence level, we observe hundreds of genes with effect sizes smaller than

0.5 in at least 20 cell types. We included this analysis in Figure S6. We have also added the probability of an effect size less than 0.5 as a column in Data S1.

**Figure S6. Number of genes with small effect sizes across cell types.** We computed the probability that the absolute logFC difference in gene expression between humans and chimpanzees was less than 0.5. We removed genes that had an effect size larger than 0.5 in any cell type (*p* ≤ 0.05). We show how the number of genes with small effect sizes varies with the confidence in this estimate as well as the number of cell types in which the effect size is small.

*Do we expect this under evolutionary models of gene regulation and species divergence, or is there a point to be made here about statistical and biological importance?*

If negative selection is the dominant force acting on gene expression levels, then genes that do not show changes in gene expression in any context should be enriched for genes that are under high degrees of selective constraint. Thus, conserved gene expression levels are a point of biological importance.

*The paper does not mention the distribution of log FC magnitudes at any point; what does this curve look like? Different by tissue?*

We now directly state the range of effect sizes (page 8) and we have also included two new figures that show the distribution of effect sizes in the complete dataset (Figure S3) and each cell type individually (Figure S4).

**Figure S3. Volcano plot of DREAM statistics.** The nominal p-value is plotted as a function of effect size estimate (log2 fold-change gene expression) for all tests across all cell types. We controlled FDR at a 5% level within each cell type. Tests that are significant at this level are colored red. The distribution in effect sizes for significant tests is plotted above the volcano plot.

**Figure S4. Ridge plot showing distribution of effect sizes in each cell type.** The density in the effect size estimate (log2 fold-change gene expression computed with DREAM) for DE genes is shown in each cell type. We excluded cell types with fewer than 10 DE genes.

We did not compare effect size distributions across cell types because this analysis would be confounded with statistical power and sampling depth.

We have also added figures showing how effect size varies with LOEUF, dN/dS, and coding identity. We found clear and consistent correlations for these statistics and we have included this as Figure S5. These results are cited on page 9.

**Figure S5. Association of logFC with LOEUF, dN/dS, and percent coding identity.** For each gene we calculated the mean absolute log2 fold-change in expression. This change significantly associates with **(A)** LOEUF decile (ρ = 0.16; *p* < 2 x 10^-16^), **(B)** dN/dS decile (ρ = 0.15; *p* < 2 x 10^-16^) and **(C)** percent coding identity decile (ρ = -0.066; *p* = 2.46 x 10^-11^).

*Major Comment 2*

In their second major comment, reviewer 1 states:

*Do the patterns of tissue specificity make sense, evolutionarily speaking? Some of the clusters in 2A are 'nice' biologically, in that they seem to impact similar related cells (eg 7, 13), but others seem noisier and messier. Mesendoderm and ectoderm mostly separate from each other, but not fully. In addition, is there anything to be gained by comparing the results here with DE result obtained from previous studies of chimpanzee/human differences in post mortem tissue?*

We highlighted the clusters of genes that are DE in all or no cell types because the evolutionary implications are straightforward. For others, there are often biological explanations for the more complex pattern of tissue-sharing. For instance, cluster 5 is highly enriched for cell cycle genes. This motif is DE in the majority of cell types, but is not DE in more mature cell types that may not be rapidly dividing, such as neurons. Differences in cell cycle are well-established in neural progenitors (for instance Mora-Bermúdez 2016 and Boyd 2015) and may extend to other cell types as well. We have now included the top 10 GO terms for each motif in Table S5.

We have not made direct comparisons to previous studies of chimpanzee/human differences, and as the reviewer asks – we are not certain what we would seek to gain from such comparisons, which will always be confounded by several factors that are difficult to resolve. Indeed, to make comparisons to datasets from different tissues we would need to make comparison to results generated in different labs, using different chimpanzee individuals, and using different technologies. The difficulty in obtaining multi-tissue samples from the same individual, with uniform processing, is precisely the reason we sought to develop a cell culture model. In short, and to be candid: Such a comparison will require quite a bit of work, questions about the validity of the comparison will remain because batch effects will be impossible to correct for, and yet we are uncertain what insight it would provide. We hope the reviewer will concur.

*Major Comment 3*

In their third major comment, reviewer 1 states

*Although the authors repeatedly mention that cell numbers are similar/roughly comparable/consistent across species, I don't always agree, and these claims are never properly tested. In Table S1 cell numbers seem consistently sparser for chimps than*

*humans, in Table S2/pg 7 line 19 6/11 clusters have fairly different cell numbers, and in Table S3, pg 7 line 46 there's clear differences in abundance in some cell types, in particular the abundance of undifferentiated cells. Is there a role for differences in make up/cell crosstalk in driving observed DE differences? I would find their claims more reassuring if they were backed up.*

This is a good point. It required additional analysis:

To measure how much cell type composition affects DE measurements, we reran our DE model, this time including cell type composition as a covariate. In order to reduce the number of covariates in our model, we performed PCA on the matrix of cell type proportions for each sample. The first two PCs explained 66% and 22% of the variance in cell type composition, respectively (88% total). We then used these PCs as covariates in our linear mixed model.

We were unable to run this analysis for 7 of the 72 cell types due to high collinearity. For the remaining 65, we used VarianceParition to estimate the variance explained by each variable in the model. For genes that we previously called as significantly expressed (DREAM 5% FDR) we show (next page) that the median percent of variation explained by PC1 is 2%, and PC2 is 1.3%. For the same gene set, we also show that the effect size estimate changes very little, with a correlation coefficient of *r* of 0.99. We included these results in Figures S9A-B.

Thank you for asking about this issue. The paper is stronger with this additional analysis.

**Figure S9. Effect of cell type proportion.** We ran a linear mixed model including two cell type proportion PCs in DREAM. We then assessed the effect of these PCs on genes with significant DE in the model that did not include cell type (5% FDR). **(A)** The median variance explained across genes in each cell type. (**B**) The effect size estimate in models with (*x-*axis) and without (*y-*axis) cell type proportion PCs.

*Minor Comment 1*

*Pg 6, line 43: "We also included a single replicate of EBs from three additional human lines of European ancestry to confirm that inter-species differences are not population- specific." Where are the results of this analysis?*

We had included this analysis but failed to cite it in the main text. In Figure S2C we use VariancePartition to estimate the percent variance explained by various factors across the entire dataset at course clustering resolution. We called the relevant factor “donor.celltype” to separate iPSCs generated from YRI LCLs from CEU Fibroblasts. The median variance explained by this factor is less than 1%. We have added a line to the text which references this figure panel and updated the figure legend to make the definition of donor.celltype clear.

**Figure S2C**. The percent of variance explained by biological and technical factors using the VariancePartition R package. Donor cell type specifies whether the iPSCs were derived from YRI LCLs or CEU fibroblasts.

*Minor Comment 2*

*pg 8, line 44 onwards: References to Fig 2 panels are incorrect through this section, eg pg 8, line 56 and pg 9, line 4.*

We have fixed the figure such that the panels and labels are in the order they were cited in the text.

*Minor Comment 3*

*pg 15, line 53: The Barreiro paper doesn't test that chimpanzee cells resist progression from HIV to AIDS. Is there a citation missing here?*

We changed this to a citation to Greenwood 2015.

*Minor Comment 4*

*pg 19, line 14: There's a lot of detail here on the sequencing and collection set up, but I actually have no idea of what replicates and lanes are associated with what library? Is this info somewhere else? If so, a call out here would be nice.*

We have updated the methods section to add clarity and avoid confusion between lanes on the 10X chip, lanes on the sequencing flowcell, and libraries. Each 10X chip lane yields one library. The libraries 1-9 are from 10X lanes 1-9 from the first replicate.

*Minor Comment 5*

*pg 20, line 11: "Finally, we used BLAT to the map the" should be "to map the"*

We have removed the extra “the”

*Minor Comment 6*

*pg 21, line 37: "a substantial number of cells" how many?*

We now state: “This resulted in a substantial proportion of cells (47%) that were assigned to hESCs…”

*Minor Comment 7*

*pg 24, line 55: "LOEUF was obtained from a study of 141,456 human genomes (Karczewski et al., 2020)." Genomes should be 'exomes'; gnomAD v2 contains 125,748 exomes and 15,708 genomes, v3 ~75k genomes.*

We have replaced the word “genomes” with “exomes”.

*Minor Comment 8*

*pg 25, line 39: "We manually annotated this set of 179 motifs to identify the TF that binds to each motif." How? (One sentence is probably enough explanation here)*

The TF or TF family that binds each motif, if known, is provided in the msigDB description for each motif. It now reads “Using their description on msigDB, we manually annotated…”

*Minor Comment 9*

*pg 58, line 58: "The inferred germ later of this cell type." later should be layer.*

We have corrected this typo.

# Reviewer 2

*Major Comment 1*

In their first major comment, reviewer 2 states:

*Authors claim that genes showing differential expression in cis to have more divergent sequences and possibly relaxed evolutionary constraints. However there exist a possibility that the more divergent genes are more likely to experience some systematic alignment bias that may skew the apparent relative expression difference between species, particularly considering that samples may have considerable sequence polymorphisms relative to reference genomes and that about 5% of chimpanzee genome sequence might not yet be accounted for, but might be generating reads. Authors should perform alignments with simulated reads of various quality to determine to what extent this can create systematic DE skew. Also reporting explicitly distribution of fold-changes would be helpful interpreting these results - how large are effects? The z-scores read from heatmap appear to be contained within (-1.5,1.5) range so not very large. With powerful statistical analysis comes risk of detection of smaller and possibly unrelated effects such as subtle alignment bias.*

We have divided our response into two sections:

*Effect size magnitude*

We are sorry this was unclear: The effect sizes are not contained in the range (-1.5 to 1.5). The heatmap with expression z-scores (Figure 2H) is showing the expression of gene sets in some cell types relative to others, with the purpose of showing that patterns of differential expression don’t necessarily correspond one-to-one with patterns of expression. To help clarify this we have modified the figure and legend describing this panel.

Additionally, we have added numerous references to the effect size distribution. We now state in the main text that absolute effect sizes range from -8.9 to 8.4, and we have added two supplementary figures that show the overall distribution of effect sizes and the distribution across cell types (Figure S3 and S4).

**Figure S3. Volcano plot of DREAM statistics.** The nominal *p*-value is plotted as a function of effect size estimate (log2 fold-change gene expression) for all tests across all cell types. We controlled FDR at a 5% level within each cell type. Tests that are significant at this level are colored red. The distribution in effect sizes for significant tests is plotted above the volcano plot.

**Figure S4. Ridge plot showing distribution of effect sizes in each cell type.** The density in the effect size estimate (log2 fold-change gene expression computed with DREAM) for DE genes is shown in each cell type. We excluded cell types with fewer than 10 DE genes.

*Alignment bias*

This was not easy because we did not find any publicly available read simulators that would simulate reads with 10X positional biases from the same null model in both species. Reads from 10X single-cell technologies are distributed over the last few hundred bases of the transcript. We had to develop something ourselves, and we acknowledge that it is not ideal, but properly developing this tool is a project of quite significant scope.

In order to assess the alignment bias of this region we generated 90bp sequences (the length of our reads) spanning the last 500bp of each gene in each species. We then mapped these sequences to the respective genomes with STAR using the same parameters used by 10X cellranger. We calculated the percent of sequences that mapped back to their original location in each species. Finally, we calculated the difference in this percent between humans and chimpanzees for each gene.

In order to assess whether alignment bias could explain the differences seen between *cis* and *trans* genes, we repeated analyses using only genes that have 0 alignment bias in the last 500bp. The association between *cis* genes and measures of relaxed evolutionary constraint are robust to alignment bias, as shown in the plot below.

Figure 4A-C shows that genes with a high *cis* proportion show evidence of relaxation of constraint. Here, only genes with 0 alignment bias in in the terminal 500bp are plotted. Boxplots show (**A**) the distribution of percent coding identity, (**B**) dN/dS, or (**C**) LOEUF for genes with a high, moderate, or low *cis* proportion. The difference between *cis* and *trans* DE is significant in each case (Wilcoxon rank-sum; *p* = 4.6 x 10^-6^, *p* = 0.026, and *p* < 2.6 x 10^-16^ respectively).

*Major Comment 2*

In their second major comment, reviewer 2 states:

*A major source of strong correlated DE between most cell types could come from uneven differentiation potential of the cell lines - either inherently due to the species differences or due to technical issues with tissue culture that has not been optimized for the chimp cells. Such possibility is suggested by a latter section of the manuscript where authors analyze tetraploid lines which are internally controlled against such effects (and then claim massive trans-environment effects (fig 3B)) and also by quite dramatic differences in relative cell type frequencies between species, which are somewhat unexpected (fig 1D vs 1E, and the supplementary tables). Indeed, a trans effect that would create such correlated changes across cell types would likely originate from a shared stimulus, such as a metabolic effect. I may have missed it but what are the GO categories of genes in motif 11 (shared DE effect).*

*The authors swept multiple potential confounding sources of DE into one label of "trans". This needs to be explicitly acknowledged in the paper and ideally, an attempt at separating these different sources of variation should be made. There are at least three sources of trans-effects here: 1) trans-regulatory environment - differences in sequences and molar concentration of proteins within cells of different species; 2) observed differences in frequencies of cell types within embryoid bodies that create a different signaling and metabolic environments, which affect expression in cell-autonomous and non-autonomous ways; 3) possibly suboptimal culture conditions for at least one species (which may also may be related to the second source of variation); in addition there might be additional effects due to derivation and epigenetic differences between the lines. In my opinion*

*authors should try to disentangle some of these effects, and in the absence of it, authors should properly discuss the nuances of the "trans" effects and limitations of the analysis in these respect. In particular, the type-2 trans effects can be accessible if the individual embryoid bodies are identifiable in sequencing (though depending on the technicalities that information could have been lost). It is also not clear to me why the tetraploid set was analyzed separately and seemingly with somewhat different methodology. Surely combining the data sets might provide a stronger more robust analysis.*

We have divided our response into several sections below:

*The effect of cell type proportion*

We have now added an analysis to explicitly test whether uneven differentiation potential explains the observed differences in DE. We generated a matrix of the relative cell type proportion of the 86 cell types in each sample, then we ran PCA on this matrix. The first two PCs explain 88% of the variation in cell type proportion (66% and 22% respectively) so we used these as covariates in our linear mixed model. We were able to run this analysis for 65 cell types (seven cell types were excluded due to high collinearity).

In order to determine whether these covariates biased our significant genes, we examined the new model parameters for the genes we previously called significant at 5% FDR. Using VariancePartition, we found that the first cell type proportion PC explained a median of 2% of the variance, while the second explained 1.3%. We also examined how the species effect size estimate changed when we included cell type proportion covariates. We found that the correlation coefficient was 0.99. Collectively, this shows that cell type composition has a minimal effect on our estimates of DE effect size and our estimates of *cis* and *trans* proportion. These new analyses are now presented in the Figure S9 and described in Materials and Methods.

**Figure S9. Effect of cell type proportion.** We ran a linear mixed model including two cell type proportion PCs in DREAM. We then assessed the effect of these PCs on genes with significant DE in the model that did not include cell type (5% FDR). **(A)** The median variance explained across genes in each cell type. (**B**) The effect size estimate in models with (*x-*axis) and without (*y-*axis) cell type proportion PCs.

*Metabolic effects*

We generated and cultured human and chimpanzee EBs under identical conditions. If these conditions are suboptimal for one species, or otherwise leads to different concentrations of

molecular species in the media, we consider these to be *bona fide* differences between the species in the context of this experiment.

Ideally, we would culture EBs that contain cells from both species. This would guarantee that cells share the same molecular environment and would lead to a more restrictive definition of *trans* effects. Unfortunately, under this setup it would have been impossible to guarantee an even representation of each species in the final collection. Differences in cell cycle length of various progenitors has already been well established, and small differences in growth and survival could lead to large differences in cell numbers over three weeks of cell culture.

*Effects due to derivation and epigenetic differences between lines*

Differences between individual lines can be a substantial source of experimental variation. It is precisely for that reason that we use biological replicates. Our group has previously reported that this is not a significant source of variation (Burrows 2016). Additionally, in replicate 1 we included EBs generated from three human iPSC lines that were derived from the same cell type, using the same methods, in tandem with the chimpanzee lines here (Gallego Romero 2015). We show in Figure S2C that the median variance explained by this factor is less than 1%.

**Figure S2C**. The percent of variance explained by biological and technical factors using the VariancePartition R package. Donor cell type specifies whether the iPSCs were derived from YRI LCLs or CEU fibroblasts.

*The nuance of ‘trans’*

Yes, this is correct. We have now modified the text to be clearer about the sources of variation that get lumped into our estimate of the effects due to *trans*.

*Separate analyses for diploid and tetraploid cells*

We used different methods to take advantage of the paired nature of hybrid measurements. By making allele-specific comparisons within single cells we can better account for technical sources of variation relative to bulk analysis. This also improved our power. Furthermore, the test was non- parametric and did not require filtering the gene-set based on expression. This meant we could test every gene that was previously tested in diploid analyses.

*Where are GO categories for motif 11*

We have now added the top 10 GO categories for each motif as supplementary table S5.

*Minor Comment 1*

*The use of "motif" for cormotif gene sets is unfortunate - I know it comes from the package terminology - but it might be confused with sequence motifs, which are also considered in the manuscript.*

We agree this can be confusing. We have replaced the word “motif” with “cormotif” when referencing genes sets from the cormotif package.

*Minor Comment 2*

*The manuscript text/figures requires some polishing - there appears to be discrepancies between text and figures, for instance panels 2E and 2F are switched in the text relative to figure, with somewhat different p value given.*

We have flipped the order of panels 2B-F such they are in the proper order relative to the text. We have corrected the p-value in the text to the correct one present in the figure.

**Second round of review**

**Reviewer 1**

I thank the authors for addressing my questions. I am satisfied by how they've chosen to handle some of the more open-ended comments I made and appreciate the more focused replies to more tractable points. As such, I have no additional comments.

**Reviewer 2**

I am satisfied with revision and recommend publication.
